# The novel influenza A (H1N1) virus pandemic: An update

**DOI:** 10.4103/1817-1737.56008

**Published:** 2009

**Authors:** N. Petrosillo, S. Di Bella, C. M. Drapeau, E. Grilli

**Affiliations:** *2^nd^ Infectious Diseases Division, National Institute for Infectious Diseases, “Lazzaro Spallanzani,” Rome, Italy*

**Keywords:** Epidemiology, H1N1, influenza

## Abstract

In the 4 months since it was first recognized, the pandemic strain of a novel influenza A (H1N1) virus has spread to all continents and, after documentation of human-to-human transmission of the virus in at least three countries in two separate World Health Organization (WHO) regions, the pandemic alert was raised to level 6. The agent responsible for this pandemic, a swine-origin influenza A (H1N1) virus (S-OIV), is characterized by a unique combination of gene segments that has not previously been identified among human or swine influenza A viruses. As of 31^th^ July 2009, 168 countries and overseas territories/communities have each reported at least one laboratory-confirmed case of pandemic H1N1 infection. There have been a total of 162,380 reported cases and 1154 associated deaths. Influenza epidemics usually take off in autumn, and it is important to prepare for an earlier start this season. Estimates from Europe indicate that 230 millions Europe inhabitants will have clinical signs and symptoms of S-OIV this autumn, and 7–35% of the clinical cases will have a fatal outcome, which means that there will be 160,000–750,000 H1N1-related deaths. A vaccine against H1N1 is expected to be the most effective tool for controlling influenza A (H1N1) infection in terms of reducing morbidity and mortality and limiting diffusion. However, there are several issues with regard to vaccine manufacture and approval, as well as production capacity, that remain unsettled. We searched the literature indexed in PubMed as well as the websites of major international health agencies to obtain the material presented in this update on the current S-OIV pandemic.

## Introduction

In recent years, there has been much concern about the possibility of an influenza pandemic caused by a novel, highly virulent, strain to which the population has no immunity and which, therefore, may be associated with high mortality rates. Social and scientific reaction toward such an event has been, as demonstrated in the case of avian flu (H5N1), institution of epidemiological surveillance and ensuring clinical preparedness.[1,2]

## The Novel Epidemic: Etiology and Hypothesis on Its Origin

The influenza pandemic the world was waiting for may have arrived on April 2009, although to date its virulence appears to be no greater than that of seasonal influenza. Mexico was the first country where there was a sharp increase in reports of patients requiring hospitalization for pneumonia and an unusual series of deaths, leading to the suspicion that a new influenza virus strain was in circulation. During the same period, officials at the Centres for Disease Control and Prevention (CDC), Atlanta, uncovered two cases of influenza, the so-called ‘swine influenza,’ that were clearly due to a novel strain; the two patients were children living in neighbouring counties in California.[3]

The causal agent, in fact, is a swine-origin influenza A (H1N1) virus (S-OIV) that is characterized by a unique combination of gene segments that has not been previously identified among human or swine influenza A viruses. Actually, the new H1N1 virus appears to be a mixture of avian, porcine, and human influenza RNA. Genomic analysis indicates that it is closely related to common reassortant swine influenza A viruses that have been isolated in North America, Europe, and Asia in the last 20 years.[4–6]

Historically, there have been four to five pandemics of influenza during the 20^th^ century, which have occurred at intervals of 9–39 years. The 1918 pandemic, caused by worldwide spread of a human influenza A (H1N1) virus, was responsible for 40–50 million deaths. An estimated 4.9 million excess deaths, representing 2% of the population, occurred in India alone.[7] After the pandemic subsided, sporadic cases of human influenza H1N1 continued to occur worldwide. H1N1 then mysteriously disappeared in 1957, likely due to both competition with the emerging pandemic H2N2 strain as well as the development of immunity to H1N1 among populations.

On January 1976, an outbreak of a respiratory disease occurred among soldiers in an army base in Fort Dix, New Jersey. Two hundred and thirty individuals had serological evidence of infection and there was one death. A novel virus H1N1 A/New Jersey/76 was identified as the cause of the epidemic that, fortunately, did not extend outside the base.[8]

In November 1977, another H1N1 strain re-emerged in the former Soviet Union, Hong Kong, and North-Eastern China. It caused a relatively mild disease, mostly in young people.[9,10] Genetic studies showed that the virus causing the 1977 epidemic was similar to the one isolated in the year 1950, but significantly different from the influenza A (H1N1) strains isolated in 1947 and 1957. Thus, it is likely that the 1977 strain was one that had been preserved since 1950.[10] The cause of the re-emergence was hypothesized to be due to accidental release from a laboratory, in the face of a waning population immunity to H1N1.[11]

The current S-OIV lineage carries three gene segments that share, with the human seasonal virus, a common descent from the 1918 H1N1 virus. Indeed, whereas 1918 influenza A (H1N1) virus likely emerged simultaneously from birds to humans and swine, S-OIV probably emerged from swine to humans. This was likely the result of a reassortment between two influenza A (H1N1) swine viruses. These two viruses were actually the products of at least four independent avian-to-mammalian cross-species transmission. During this process of evolution there were at least four reassortments of gene segments among avian, human and swine-adapted viruses.[12]

It is not clear, however, whether this sharing of genes segments between human seasonal influenza A (H3N2) and current seasonal influenza A (H1N1) will allow S-OIV-infected individuals to develop effective immunity and clinical protection against S-OIV itself.

The aim of this review is to give an update of the global situation of the 2009 S-OIV pandemic. To obtain the material presented in this article, we searched the PubMed database using the following key words: *H1N1, S-OIV,* and *swine flu.* We also visited the websites of the main international health agencies, namely the World Health Organization (WHO) and Centres for Disease Control (CDC), Atlanta, to search for relevant matter. The cut-off date for the search was August 10, 2009.

## Global Epidemiology

According to the WHO,[13] all continents have been affected by the pandemic. As of 31^st^ July 2009, 168 countries and overseas territories/communities have each reported at least one laboratory-confirmed case of pandemic (H1N1) influenza, with a total of 162,380 reported cases and 1154 associated deaths. However, the number of cases reported actually understates the real number of cases, given that countries are no longer required to test and report individual cases.

### Estimates of the impact of the pandemic

In Europe a major wave of the S-OIV epidemic is expected this autumn;[14] this is also likely in other countries that experienced the first wave in May–July. However, it is not possible at this point to make any prediction regarding the possible infection rates and the timing of the epidemic in the different countries.

Influenza epidemics usually take off in autumn, and it is important to prepare for an earlier start in this season. In the UK, which was one of the countries to suffer the first wave of the S-OIV epidemic, the Department of Health estimates that 30% of the general population will develop clinical disease, with 15% suffering complications and 2% requiring hospitalization. The case fatality rate is expected to be 0.1–0.35% of the clinical cases. In the UK, the expected rate for sickness work absence is 12% of workforce at the peak level.[15]

Europe has nearly 700 million inhabitants. If the estimates from UK apply in the rest of the continent, 230 millions European inhabitants will report clinical signs and symptoms of S-OIV infection this autumn, and there will be approximately 230,000–750,000 related deaths, i.e., about 33–107 deaths per 100,000 inhabitants. If the case fatality rate from the WHO S-OIV data (1154 deaths/162,380 cases, i.e., 0.07% of clinical cases) is applied the number of deaths could be lower, i.e., around 160,000.

During years 1972–1992, influenza-related mortality and hospitalization rates in the US were, respectively, 9.1 and 50 per 100,000 population.[16] A possible explanation for the difference between the 1972–1992 US rate and the UK estimates for autumn, 2009, could be the difference in age structure in the previous period and in the current one due to aging of the population. In fact, in the period 1972–92 during the inter-pandemic years, over 90% of influenza-related deaths occurred among persons 65 years of age or older; consequently, the incidence of influenza-related mortality was 100-fold higher for these age-groups than among younger persons. The higher UK fatality rate estimate can be explained by the fact that there has been a substantial increase in the number of persons aged ≥65 years and these persons are at increased risk for death from influenza complications. Indeed, though the rates of infection are highest among children and young adults, complications, hospitalizations, and deaths from influenza are higher among persons aged ≥65 years and those (of any age) who have medical conditions that place them at increased risk.

## Transmission Routes of S-OIV

From what has been documented in international public health reports, it is clear that human S-OIV infection spreads easily through different countries and continents. The emergence of S-OIV infection among humans represents the greatest pandemic threat ever observed since the emergence of an (H3N2) influenza virus in 1968.[5]

Influenza due to a virus of swine origin has been previously reported only sporadically in humans, and generally affected young persons and their family members with recent exposure to pigs.[17] Some cases with no history of exposure to pigs have also been reported[18,19] and, although these reports are quiet scant, it gives credence to the hypothesis that human-to-human transmission is possible.

Since its first recognition, the pandemic strain of S-OIV has spread worldwide within 3 months. After human-to-human transmission was documented in at least three countries of two WHO regions, the pandemic alert was raised to phase 6 on 11^th^ June, 2009.

The transmission routes and incubation period of S-OIV are similar to that of any other influenza virus.[20,21] The influenza virus belongs to the Orthomyxoviridae family, has an envelope, and has a size of 0.08–0.12 μm. The main route of transmission is respiratory, through inhalation of large droplets and, possibly, small droplet nuclei[22] expelled while coughing or sneezing. Transmission via respiratory droplets requires close contact between source and recipient because droplets do not remain suspended in air for long and generally travel only short distances (<6 ft). Transmission on the hands of patients and their caregivers has also been described as being potentially relevant.[23]

Another potential transmission routes is contact with fomites[24] contaminated with respiratory or gastrointestinal fluids.[25,26] Since many patients with S-OIV infection have complained of diarrhea,[27,28] feco-oral transmission should be considered a potential transmission route,[22] until further data are available.

In the early epidemic phase, most of the cases were imported, whereas currently the majority of cases are autochthonous. For instance, recent German surveillance reports for S-OIV notified 105 domestic cases, i.e., 53% of the total cases reported from the country. For 96 out of 105 domestic cases (91%), the source of infection was known; moreover, 73 of the 96 cases (76%) were outbreak-related, while 23 cases were related to an imported case.[29]

Schools represent the ideal environment for influenza virus transmission because of crowding and close contact between children, who usually share items and toys. Indeed, school clusters have been described everywhere.[29,31] All events involving crowds – like the case of the rock festival in Belgium[32] or private parties[29] – have the potential to cause the occurrence of influenza clusters. Table 1 shows some recommendations for minimizing the diffusion of S-OIV in community settings[33].

**Table 1:** Recommendations for minimizing the diffusion of S-OIV in the community-settings

Persons with influenza-like illness should be advised to stay home for 7 days after the onset of illness or at least 24 h after symptoms have resolved.
Persons who are at high risk of complications from S-OIV should consider the risk of exposure to S-OIV if they attend public gatherings where the virus is circulating and, possibly, should consider staying away from public gatherings.
At public gatherings hand washing facilities (with soap and running water, hand sanitizer, and tissues) should be widely available. On-site medical assessment and care for persons with influenza-like illness should be provided.
At public gatherings information on S-OIV transmission and on the ways to reduce the risk of acquiring infection should be widely provided.

Adapted from reference 33

## Clinical Presentation

### Symptoms and signs

Influenza is the most frequent cause of acute respiratory illness requiring medical intervention.[34] It usually causes an acute, self-limiting, febrile illness but it can also lead to severe complications or death, particularly in patients with compromised immunity (e.g., pregnant women, elders, HIV-infected persons, etc.) or underlying medical conditions (e.g., cardiac or pulmonary disease).[35] The pandemic is still under study but, so far, the clinical presentation of the novel S-OIV A (H1N1) seems to resemble that of seasonal influenza, and the majority of patients infected with the pandemic virus experience mild symptoms and recover fully within a week, even without medical treatment.

In order to better define the clinical features of this pandemic, we reviewed the descriptions of S-OIV cases in the literature from across the world [Tables 2 and 3]. According to this data, S-OIV patients complained mostly of the classical influenza symptoms; in addition, S-OIV patients also often have diarrhea and vomiting, which are not usually seen in seasonal influenza.[5] Fever was reported by a median of 87% (62–100%) of cases, cough by 82.5% (59–100%), sore throat by 57% (2–82%), diarrhea by 13.5% (2–50%), and vomiting by 12.5% (2–50%) [Table 2]. Other symptoms reported by patients include myalgia, arthralgia, nasal congestion, headache, anorexia, sneezing, nausea, shortness of breath, and conjunctivitis.

**Table 2:** Epidemiological and clinical characteristics of S-OIV* patients worldwide, as of August 10,2009

Reference	Geographic area	S-OIV cases	Age		Main clinical symptoms – no/total no (%)				
				
			Median	Range	Fever	Cough	Sore throat	Diarrhea	Vomiting
3	California, US	2 children	9.5	9-10	2/2 (100)	2/2 (100)	-	-	1/2 (50)
36	California and Texas, US	5	41	16-54	3/3 (100)	2/3 (67)	1/3 (33)	1/3 (33)	1/3 (33)
28	New York, US	44 in a school outbreak	15	14-21	42/44 (96)	43/44 (98)	36/44 (82)	21/44 (48)	-
27	Mexico	97		<1-59	15/16 (94)	13/16 (81)	-	5/16 (31)	5/16 (31)
37	Mexico	949			56/57 (98)	49/52 (94)	-	-	-
		22 hospitalized patients	-	-					
	US	642	-	<1-81	262/292 (90)	249/296 (84)	176/290 (61)	65/249 (26)	54/221 (24)
		35 hospitalized patients	15	<1-53	-	-	-	-	-
	Other countries	309	27.1	2-62	-	-	-	-	-
38	Spain	98	22	14-55	87/91 (96)	83/87 (95)	29/48 (60)	17/41 (41)	4/32 (13)
39	US	3 pregnant women	33	29-35	3/3 (100)	3/3 (100)	2/3 (67)	1/3 (33)	-
40	California, US	30 hospitalized patients	27.5	27-89	29/30 (97)	23/30 (77)	10/30 (33)	3/30 (10)	14/30 (46)
41	Greece	2	20.5	20-21	2/2 (100)	2/2 (100)	-	-	-
42	France	16	29	<1-65	10/16 (62)	16/16 (100)	8/14 (57)	2/16 (13)	1/16 (6)
43	UK	252	12	0-73	–(92)	–(40)	–(79)	–(27)	–(33)
44	EU	1128	23	<1-73	290/371 (78)	248/371 (67)	172/371 (46)	45/371 (12)	49/371 (13)
45	Japan	49 in an outbreak	17	5-60	43/49 (88)	38/48 (79)	35/49 (71)	7/49 (14)	6/49 (12)
5	US	642	20	<1-81	371/394 (94)	365/397 (92)	242/367 (66)	82/323 (25)	74/295 (25)
46	Japan	401	16	1-69	206/217 (95)	128/217 (59)	85/217 (39)	13/217 (6)	-
47	Mexico	18 hospitalized patients with pneumonia	38	<1-61	18/18 (100)	18/18 (100)	-	4/18 (22)	-
48	Singapore	10 hospitalized patients	28	18-43	9/10 (90)	7/10 (70)	3/10 (30)	0/10 (0)	-
49	UK	63	-	13-18	39/63 (62)	-	34/63 (54)	diarrhea/vomilting	3/63 (5)
50	Italy	158	28	0-69	72/86 (84)	60/86 (70)	35/86 (41)	8/86 (9)	6/86 (7)
51	UK	64 in an outbreak	g†	4-12†	54/64 (84)	-	38/64 (59)	14/64 (22)	-
52	Netherlands	115	-	-	76 (88)	-	-	9 (9)	-
53	Belgium	43	28	<1-51	36/42 (86)	40/42 (95)	1/42 (2)	5/42 (12)	1/42 (2)
54	Michigan, US	10 patients in ICU‡	46	21-53	7/10 (70)	7/10 (70)	2/10 (20)	1/10 (10)	2/10 (20)
55	Canada	42	-	12-46	–(59)	–(90)	–(76)	-	-
31	France	358	23	<1-77	286/333 (86)	294/336 (88)	72/323 (22)	14/324 (4)	18/328 (5)
30	Japan	105 (secondary school)	16	13-53	94/105 (90)§	86/104 (83)	68/104 (65)	19/96 (20)	5/94 (5)
		7 (elementary school)	11	9-12	7/7 (100)	7/7 (100)	5/7 (71)	1/7 (14)	0/5 (0)
		59	15	6-48	-	-	-	-	-
56	Greece	312	-	-	235/277 (85)	224/274 (82)	110/269 (41)	20/266 (8)	14/263 (5)
57	US	34 pregnant women	26	15-42	33/34 (97)	32/34 (94)	17/34 (50)	4/34 (12)	6/34 (18)
58	Colombia	183	27	0-72	153/180 (85)	176/181 (97)	133/177 (75)	10/180 (6)	-
29	Germany	198	18	1-67	–(82)	–(69)	–(17)	–(2)	-(1)
32	Belgium	12 (outbreak in a rock festival)	20	18-45	11/12 (92)	12/12 (100)	-	1/12 (8)	1/12 (8)
59	China	3	19	18-30	3/3 (100)	2/3 (67)	3/3 (100)	0/3 (0)	0/3 (0)

* Swine-Origin Influenza A (H1N1) Virus,

† Age range and median calculated excluding 2 members of the staff,

‡ Intensive Care Unit,

§ High grade fever of or above 38°C

### Age

The younger age-groups appear to be much more susceptible than the elderly to S-OIV infection. This is concordant with the age-stratified sero-epidemiological data, which suggests that persons >60 years of age are more likely to have neutralizing antibodies to the virus.[60] According to a report of 642 confirmed cases in the US, with patients ranging in age from 3 months to 81 years, 60% of patients were ≤18 years old, 40% were 10–18 years old, and only 5% were ≥51 years of age.[5] The majority (>97%) of confirmed cases of S-OIV infection in Mexico were also in those <60 years of age.[61]

### Hospitalization

One of the more controversial issues is the question of hospitalization for S-OIV infection; hospitalization is often deemed necessary because of the need for isolation of these patients. During the influenza seasons of 1972–1992, the average seasonal burden due to influenza was estimated to be about 50 hospitalizations per 100,000 US inhabitants per season.[16]

From March 28 to May 4, 35 out of 642 confirmed cases (5%) in the US and 52 out of 949 cases (6%) in Mexico were hospitalized.[37] In Europe, the initial approach was different from that in North America, with some countries placing the emphasis on case finding and contact-tracing, with antiviral treatment of patients and chemoprophylaxis of contacts. Later, cases were isolated in hospitals and quarantine was practised in most European countries though not in the UK.[2]

In this phase of the pandemic, most national and international guidelines recommend that individuals with suspected flu be treated at home so as to avoid contact with other people. In case of any of the emergency warning signs, which includes difficulty breathing, confusion, severe or persistent vomiting, and worsening of cough, urgent medical attention is required. Patients who present with flu-like symptoms and then improve, only to return with fever and a worse cough than before, should be suspected to have respiratory complications. For patients at high risk for developing influenza complications (see below), hospital admission should be considered.

In the case of hospital admission, patients who are confirmed, probable, or suspected cases should be placed directly into individual rooms and the door should be kept closed. Healthcare personnel interacting with these patients should adhere to the guidelines for proper hand hygiene. Nonsterile gloves and gowns should be donned, and eye protection and respiratory protection ensured, before entering the patient's room. Isolation precautions should be continued for 7 days from symptom onset or until resolution of symptoms.[62]

## Complications

Pneumonia is the most common complication of seasonal influenza. This complication is rare in interpandemic eras but becomes more frequent when a pandemic occurs. From March 24 to April 29, 2009, a total of 2155 cases of severe pneumonia were reported in Mexico. During this period, that represented the early phase of the S-OIV epidemic, a pronounced shift in morbidity was evident, with 71% of cases of severe pneumonia occurring in patients between the ages of 5 and 59 years, as compared with an average of 32% of cases in that age-group during previous periods.[63] Similarly, there was a marked change in the mortality pattern, with an increase in the mortality rate in the 5-59 year age-group as compared with the rates observed in this same age-groups during previous periods of epidemic influenza (87% *vs* 17%).[63]

Data from the literature reported 79 cases of pneumonia [Table 3]; however, rates of pneumonia complication varied according to the population on study. Out of 642 confirmed cases of human S-OIV infection in the US, 36 (9%) required hospitalization. Of 22 hospitalized patients (on whom data was available), 11 (50%) had pneumonia, eight required admission to an intensive care unit, four had respiratory failure, and two died.[5]

**Table 3:** Hospitalization, complication, treatment and outcome of S-OIV* cases worldwide, as of August 10,2009

Reference	Geographic area	Hospitalization no./total no. (%)	Pneumonia no/total no. (%)	ICU† no./total no. (%)	Mechanical ventilation no./total no. (%)	Oseltamivir/Zanamivir no./total no. (%)	Underlying conditions no./total no. (%)	Died no./total no. (%)
3	California, US	0/2 (0)	0/2 (0)	0/2 (0)	0/2 (0)	0/2 (0)	-	0/2 (0)
36	California and Texas, US	1/5 (20)	-	0/5 (0)	0/5 (0)	0/5 (0)	1/5 (20)	0/5 (0)
28	New York, US	1/44 (2)	-	0/44 (0)	0/44 (0)	0/44 (0)	-	0/44 (0)
27	Mexico	20/24 (83)	12/15 (80)	8/16 (50)	7/16 (44)	-	3/16 (19)	7/97 (7)
37	Mexico	-	-	-	-	-	-	42/949 (4)
		All hospitalized patients	-	-	-	-	6/22 (27)	7/22 (32)
	US	35/642 (5)	-	-	-	-	-	2/642 (0)
		All hospitalized patients	-	-	-	-	-	2/35 (6)
	Other countries	4/309 (1)	-	-	-	-	-	0/309 (0)
38	Spain	-	-	-	-	-	-	0/98 (0)
39	US	1/3 (33)	1/3 (33)	1/3 (33)	1/3 (33)	3/3 (100)	2/3 (67)	1/3 (33)
40	California, US	All hospitalized patients	15/25 (60)	6/30 (20)	4/30 (13)	15/30 (50)	19/30 (64)	0/30 (0)
41	Greece	0/2 (0)	0/2 (0)	0/2 (0)	0/2 (0)	0/2 (0)	-	0/2 (0)
42	France	-	0/16 (0)	0/16 (0)	0/16 (0)	16/16 (100)	4/16 (25)	0/16 (0)
43	UK	4/252 (2)	-	-	-	-	-	0/252 (0)
44	EU	105/291 (36)	4/286 (1)	-	-	258/292 (88)	24/1128 (2)	-
45	Japan	-	-	-	0/49 (0)	48/49 (98)	9/49 (18)	0/49 (0)
5	US	36/399 (9)	11/22 (50)	8/22 (36)	4/22 (18)	14/19 (74)	12/22 (55)	2/36 (6)
46	Japan	135/217 (62)‡	0/217 (0)	0/217 (0)	0/217 (0)	∼90% of 217	6/217 (3)	0/217 (0)
47	Mexico	All hospitalized patients	18/18 (100)	-	12/18 (67)	14/18 (78)	8/18 (44)	7/18 (39)
48	Singapore	All hospitalzed patients	0/10 (0)	0/10 (0)	0/10 (100)	10/10 (100)	1/10 (10)	0/10 (0)
49	UK	0/63 (0)	-	0/63 (0)	0/63 (0)	-	-	0/63 (0)
50	Italy	22/86 (26)	-	0/158 (0)	0/158 (0)	86/86 (100)	9/80 (11)	0/158 (0)
51	UK	0/64 (0)	-	0/64 (0)	0/64 (0)	-	-	0/64 (0)
52	Netherlands	2/115 (2)	2/115 (2)	1/115 (1)	-	-	3/46 (7)	0/115 (0)
53	Belgium	1/42 (2)	-	0/42 (0)	0/42 (0)	-	-	0/42 (0)
54	Michigan, US	All hospitalized patients	10/10 (100)	10/10 (100)	10/10 (100)	10/10 (100)	10/10 (100)	3/10 (30)
55	Canada	0/42 (0)	-	0/42 (0)	0/42 (0)	42/42 (100)	-	0/42 (0)
31	France	2/358 (1)	2/358 (1)	-	1/358 (2)	-	-	0/358 (0)
30	+Japan	-	-	-	-	165/171 (96)§	-	-
		-	-	-	-		-	-
		-	-	-	-		-	-
56	Greece	-	-	-	-	-	-	0/312 (0)
57	US	34/34 (100)	4/34 (12)	3/34 (9)	1/34 (3)	17/34 (50)	9/34 (26)^II^	1/34 (3)
58	Colombia	26/183 (14)	-	-	-	-	2/7 (29)¶	7/183 (4)
29	Germany	40/198 (20)	-	-	0/198 (0)	-	4/18 (22)	0/198 (0)
32	Belgium	0/12 (0)	-	0/12 (0)	0/12 (0)	-	-	0/12 (0)
59	China	3/3 (100)	0/3 (0)	0/3 (0)	0/3 (0)	3/3 (100)	-	0/3 (0)

* Swine-Origin Influenza A (H1N1) virus,

† Intensive Care Unit,

‡ Only three cases required hospitalisation due to underlying medical conditions, althouh a total of 135 cases were hospitalized for the purpose of isolation,

§ 171 cases in Osaka,

II 7 with history of asthma (only one taking medication for it); ^1^using insulin for diabetes in pregnancy, ^1^taking labetalol for hypertension and methimazole for hyperthyroidism,

¶ Only 2 of the fatal cases (7) had underlying medical conditions, including obesity (n=1) and underweight (n=1)

In Mexico, among 98 patients hospitalized for acute respiratory illness at the National Institute of Respiratory Diseases in Mexico City during the early stages of the influenza A (H1N1) outbreak, 18 cases of pneumonia and confirmed S-OIV infection were identified. All these patients had bilateral pneumonia and had complaints of fever, cough, and dyspnea. All of them had increased serum lactate dehydrogenase levels; creatinine kinase levels were increased in 62%, and lymphopenia was present in 61%. Twelve patients required mechanical ventilation and seven died.[47] All pneumonias were radiologically confirmed, with 11 patients having bilateral patchy alveolar opacities (predominantly basal) affecting three or four quadrants.[47]

Among non-pulmonary complications of influenza are various forms of central nervous system involvement, including encephalitis, transverse myelitis, aseptic meningitis, and the Guillan-Barré syndrome.[64–68] Four cases of neurological complications associated with S-OIV infection in children have been described in the literature. These patients were aged 7–17 years and were admitted with signs of influenza-like illness and seizures or altered mental status. Three of the four patients had abnormal electroencephalograms (EEGs). In all four patients, novel influenza A (H1N1) viral RNA was detected in nasopharyngeal specimens but not in cerebrospinal fluid (CSF). All four patients recovered fully and had no neurologic sequelae at discharge. These findings indicate that, as with seasonal influenza, neurologic complications can occur after respiratory tract infection with novel influenza A (H1N1).[69]

### Risk groups

The risk of morbidity from seasonal influenza is higher among pregnant women. Pregnant women with underlying medical conditions such as asthma are at particularly high risk for influenza-related complications.[70,71] Jamieson *et al*. reported 31 confirmed and three probable cases of S-OIV infection in pregnant women. Eleven (32%) women were admitted to hospital and of them three were admitted to intensive care units (ICU). Four women had confirmed pneumonia. Of the 45 deaths from pandemic H1N1 virus infection reported to the CDC from April 15 to June 16, 2009, six (13%) were in pregnant women.[57]

WHO strongly recommends that in areas where S-OIV infection is widespread pregnant women, as well as the clinician treating them, be alert to symptoms of influenza-like illness. In addition to the increased risk for pregnant women, groups at increased risk of severe or fatal illness include people with underlying medical conditions, particularly chronic lung disease, cardiovascular disease, diabetes, and immunosuppression.

Some preliminary studies suggest that extreme obesity represents a risk factor for severe disease.[54,58] Obesity has not been identified previously as a risk factor for severe complications of seasonal influenza. In a report from Michigan on 10 ICU patients with S-OIV infection and ARDS, nine patients were obese, with seven being extremely obese (BMI ≥40); three of these patients died.[54] In another study from Colombia, seven patients died; of them, one was obese and one underweight.[58]

Finally, HIV-infected people (especially those with AIDS or low CD4 cell counts) who acquire H1N1 infection may be at increased risk for more severe disease and complications.[44] In addition, they may be at increased risk for secondary bacterial infections, including pneumonia.[72–74] In immunocompromised hosts the illness may last longer than in immunocompetent ones and the virus may replicate for weeks to months, leading to more prolonged H1N1 virus shedding.[75,76]

## Antiviral treatment

Two classes of antiviral drugs are available for the treatment of influenza: neuraminidase inhibitors, (zanamivir and oseltamivir) and adamantanes (amantadine and rimantadine).[5] Genetic analysis has shown that S-OIV is resistant to the adamantanes, whereas it is susceptible to oseltamivir and zanamivir.[77]

At the beginning of the S-OIV pandemic, the use of neuraminidase inhibitors was encouraged because oseltamivir and zanamivir have been proven to be able to reduce the duration and the incidence of complications of seasonal influenza,[78] especially when treatment is started early (ideally, within 48 h of the onset of symptoms).[79] Indeed, data from our review evidenced an overuse of oseltamivir or zanamivir. Clinical studies have shown that 88–100% patients received antiviral treatment, even though patients with complications or with underlying disease ranged from 2 to 11%.[44,46,50]

As more data on antiviral effectiveness, clinical features of illness, adverse events from antiviral use, and antiviral susceptibility become available, recommendations on treatment are changing. Until the year 2007, resistance of influenza A (H1N1) virus to neuraminidase agents occurred only in a small proportion of cases.[80] More recently, a high rate of oseltamivir resistance in seasonal influenza virus A (H1N1) infection has been reported in Europe[81] and the US.[82] Moreover, two cases of oseltamivir-resistant S-OIV in immunosuppressed patients have already been reported.[83]

Till date, the use of oseltamivir and zanamivir has been restricted to patients at relatively higher risk for influenza complications,[79] i.e., children <5 years old; adults ≥65 years; persons with conditions such as chronic pulmonary, cardiovascular, renal, hepatic, hematologic, neurologic, neuromuscular, or metabolic disorders; persons with immunosuppression due to any cause; pregnant women; residents of nursing homes and chronic-care facilities; persons (<19 years old) receiving long-term aspirin therapy.

The CDC suggest that antiviral chemoprophylaxis with either oseltamivir or zanamivir can be considered for the following[78]:

1. Close contacts of cases (confirmed, probable, or suspected) who are at high risk for complications of influenza
2. Health care personnel, public health workers, or first responders who have had a recognized, unprotected close contact exposure to a person with novel (H1N1) influenza virus infection (confirmed, probable, or suspected) during that person's infectious period.

## Vaccine Against H1N1

A vaccine against H1N1 is expected to be the most effective tool for controlling influenza A (H1N1) infection in terms of reducing morbidity and mortality and limiting diffusion. However, several issues exist around vaccine manufacture and approval, as well as production capacity.

WHO conducted a survey in collaboration with all influenza vaccine manufacturers to review the current status of northern hemisphere seasonal vaccine production, and to assess the capabilities of the manufacturers for supplying the new influenza A(H1N1) vaccine over a 1-year period. The survey showed that by the time of the expected new epidemic wave most manufacturers will be technically ready to initiate large-scale production of the pandemic influenza vaccine.[84]

However, there are still many controversies regarding the number of vaccine doses required to achieve full immunological protection and the possible utility of adjuvants.[85,86] Another critical point is related to manufacturing capacity, which is likely to be inadequate to meet the entire demand of the large number of requesting countries.

There is also great concern related to the fast-track approval processes for the H1N1 vaccine set up by many regulatory agencies since this means that a vaccine might be licensed for use without satisfying the usual safety and efficacy requirements. For instance, the emergence of swine influenza at Fort Dix in 1976 led to the implementation of a mass vaccination program, with 40 million civilian vaccinations. Following the vaccination, there were 532 cases of the Guillain-Barré syndrome (a rare side effect of influenza vaccination) and 32 deaths.[87] The mass vaccination campaign was then stopped and the vaccine was withdrawn.

Another important query is who should be prescribed the H1N1 vaccine. Seasonal influenza vaccine is currently provided to all persons who wish to reduce the risk for becoming ill with influenza or of transmitting it to others. Routine annual vaccination is recommended for high-risk groups, including children aged 6 months to 18 years, all persons aged ≥50 years, and other adults at risk for medical complications from influenza.[88] Moreover, all persons who live with or care for persons at high risk for influenza-related complications, including contacts of children aged <6 months, should receive influenza vaccine annually.

Overall, it is estimated that around 85% population is eligible for influenza vaccination, which is likely to be above the production capacities for H1N1 vaccine, at least in the early phase of a pandemic. Countries need to determine their order of priority based on country-specific conditions, in order to deliver vaccination to priority groups and to groups with a social utility.

On July 29, 2009, the Advisory Committee on Immunization Practices (ACIP) — an advisory committee to CDC — recommended that novel H1N1 flu vaccine be made available first to the following five groups in the US[89]:

- Pregnant women
- Health care workers and emergency medical responders
- People caring for infants under 6 months of age
- Children and young adults from 6 months to 24 years
- People aged 25–64 years with underlying medical conditions (e.g., asthma, diabetes)

Combined, these groups would equal approximately 159 million individuals in the US.

In conclusion, as the S-OIV pandemic spreads worldwide, we can expect a significant number of clinical cases this autumn, and there will be an obvious impact on morbidity and mortality rates. In order to minimize the public health impact and the associated social consequences, all countries should prepare their plans in time to face the epidemic appropriately in terms of clinical guidance, infection control, and hospital preparedness. To achieve satisfactory endpoints, national health organizations should provide recommendations to physicians, patients, and community settings and should also implement vaccination programmes, with a clear idea of where the priorities lie.
